# Value of Autoantibody Expression During Long-Term Follow-Up in Paediatric ALL Patients After Allogeneic Haematopoietic Stem Cell Transplantation

**DOI:** 10.3389/fped.2021.788360

**Published:** 2021-12-21

**Authors:** Anita Lawitschka, Leila Ronceray, Dorothea Bauer, Michael Rittenschober, Natalia Zubarovskaya, Rene Geyeregger, Winfried F. Pickl, Zoya Kuzmina

**Affiliations:** ^1^Stem Cell Transplantation Unit, St. Anna Children's Hospital, Medical University of Vienna, Vienna, Austria; ^2^Children's Cancer Research Institute, Vienna, Austria; ^3^Institute of Immunology, Medical University of Vienna, Vienna, Austria; ^4^Pulmonology Department Ottakring Hospital, Medical University of Vienna, Vienna, Austria

**Keywords:** paediatric, haematopoietic stem cell transplantation, acute lymphoblastic leukaemia, graft-versus-host disease, autoantibody, B cells, immune reconstitution

## Abstract

**Objectives:** Chronic graft-versus-host disease (cGvHD) following haematopoietic stem cell transplantation (HSCT) shares many similarities with *de novo* autoimmune disorders, being associated with the presence of autoantibodies. However, data on the implication of autoantibodies in paediatric HSCT recipients are scarce. In this single-centre study of paediatric patients with acute lymphoblastic leukaemia (ALL) surviving longer than 3 months, our objectives were to evaluate autoantibody expression and investigate the correlation with cGvHD and immune reconstitution using serially monitored parameters.

**Methods:** We investigated circulating autoantibodies together with cellular and humoral parameters [including major T- and B-cell subsets, natural killer (NK) cells, and immunoglobulin levels] in 440 samples from 74 patients (median age 10.9 years, range 2.7–22.2 years) serially during long-term follow-up of median 8 years (range 0.4–19.3 years). Evaluations comprised of patient and transplant characteristics, precisely reviewed details of National Institute of Health (NIH)-defined cGvHD, and outcome data such as relapse, overall survival (OS) and mortality. Analysis of these clinical parameters was performed to identify possible associations.

**Results:** Autoantibodies were detected in 65% (48/74) of patients. Anti-nuclear antibodies were the most common, occurring in 75% (36/48) of patients with autoantibodies. When comparing demographic data and transplant characteristics, there were no significant differences between patients with and without autoantibody expression; 5-year OS was excellent, at 96.4 and 95.8%, respectively. Neither the expression of autoantibodies nor the occurrence of cGvHD correlated with significantly worse OS or relapse rate. Furthermore, there was no significant association between autoantibody profiles and the incidence, overall severity or organ involvement of cGvHD. Patients with autoantibodies showed significantly better immune reconstitution, with overall higher numbers of T cells, B cells, and serum immunoglobulins. In autoantibody-positive patients with cGvHD, autoantibody production positively correlated with the expansion of CD56+ NK cells (236.1 vs. 165.6 × 10^3^ cells/mL, respectively; *p* = 0.023) and with signs of B-cell perturbation, such as higher CD21^low^ B cells (23.8 vs. 11.8 × 10^3^ cells/mL, respectively; *p* = 0.044) and a higher ratio of CD21^low^ B cells/CD27^+^ memory B cells (1.7 vs. 0.4, respectively; *p* = 0.006) in comparison to autoantibody-positive patients without cGvHD. Furthermore, when assessing the correlation between autoantibody positivity and the *activity* of cGvHD at time of analysis, indicators of aberrant B-cell homeostasis were substantiated by a lower proportion of CD27^+^ memory B cells (9.1 vs. 14.9%, respectively; *p* = 0.028), a higher ratio of class-switched CD27^+^IgD^−^/CD27^+^ memory B cells (3.5 vs. 5.1%, respectively; *p* = 0.013), significantly elevated numbers of CD21^low^ B cells (36.8 vs. 11.8 × 10^3^ cells/mL, respectively; *p* = 0.013) and a higher ratio of CD21^low^B cells/CD27^+^ memory B cells (2.4 vs. 0.4, respectively; *p* = 0.034) in the *active* vs. the no cGvHD group. We then assessed the potential role of autoantibody expression in the context of elevated CD19^+^CD21^low^ B cells (cutoff >7%), a well-known marker of cGvHD. Surprisingly we found a significant higher proportion of those cases where elevated CD21^low^ B cells correlated with *active* cGvHD in samples from the autoantibody-negative group vs. the antibody-positive group (82 vs. 47%, respectively; *p* = 0.0053).

When comparing immune parameters of the large proportion of survivors (89%) with the small proportion of non-survivors (11%), data revealed normalisation within the B-cell compartment of survivors: there were increased numbers of CD27^+^ memory B cells (54.9 vs. 30.6 × 10^3^ cells/mL, respectively; *p* = 0.05), class-switched CD27^+^IgD^−^ B cells (21.2 vs. 5.0 × 10^3^ cells/mL, respectively; *p* < 0.0001), and immunoglobulin G4 (40.9 vs. 19.4 mg/dL, respectively; *p* < 0.0001). Overall mortality was significantly associated with an elevated proportion of CD21^low^ B cells (13.4 vs. 8.8%, respectively; *p* = 0.039) and CD56^+^ NK cells (238.8 vs. 314.1 × 10^3^ cells/mL, respectively; *p* = 0.019). In multivariate analysis, better OS was significantly associated with lower numbers of CD56^+^ NK cells [hazard ratio (HR) 0.98, *p* = 0.041] and higher numbers of CD27^+^ memory B cells [(HR) 1.62, *p* = 0.014].

**Conclusion:** Our data shows that autoantibody profiles are not suitable biomarkers for diagnosing cGvHD in children or for predicting cGvHD severity, disease course and outcome. We identified a number of indicators of aberrant immune homeostasis associated with active cGvHD in paediatric ALL patients after HSCT. These findings confirm published results and suggest that candidate B cell subpopulations may serve as a surrogate measure for characterisation of cGvHD in paediatric HSCT for malignant diseases, and warrants confirmation in larger, multicentre studies.

## Introduction

Allogeneic haematopoietic stem cell transplantation (HSCT) is a potentially curative treatment for acute lymphoblastic leukaemia (ALL) in paediatric and adolescent patients. Recently, it has been shown to have excellent outcomes with low treatment-related mortality (1). However, successful long-term outcomes may be limited by chronic graft-versus-host disease (cGvHD), a serious and complex, multisystem immunological complication of HSCT and major cause of late non-relapse morbidity and mortality (2, 3). The incidence of cGvHD is ~50% in adults while the incidence in paediatric patients is lower (5–30%) (4, 5). The clinical presentation of cGvHD may resemble those seen in autoimmune disorders and nearly every organ system may be affected resulting in poor physical functioning and disability (2, 3). Regarding diagnosis and staging of cGvHD, a major advancement has been made by the publication and validation of the National Institutes for Health (NIH) Consensus Criteria in 2005 with revision in 2014 (6, 7). A paediatric adaption of the NIH documentation forms for daily clinical use has been published in the European Society for Blood and Marrow Transplantation (EBMT) handbook (3). Recent advances in understanding the pathophysiology of cGvHD demonstrated that the disease is characterised by a combination of allogeneic and autoimmune dysregulation (8) with prolonged immunodeficiency (9, 10). It is well-known that alloreactive CD8^+^ T cells play a crucial role in the development of a graft-versus-leukaemia (GVL) effect and GvHD and that, amongst other cell types, CD4^+^ T cells stimulate the production of autoantibodies after HSCT (11–13). The role of impaired B-cell homeostasis in cGvHD has been shown by many groups (9, 14, 15), in adults, and was recently observed in the paediatric population recently (16). Along those lines our centre observed that both cGvHD and its activity were associated with B-cell perturbation including low numbers of CD19^+^CD27^+^ memory B cells and increased frequencies of circulating CD19^+^CD21^low^ B cells in a paediatric population (*n* = 146) (17). Chronic GvHD shares many similarities with *de novo* autoimmune disorders: presence of autoantibodies leads to target tissue damage, immune complex formation, and tissue deposition (15). An association between cGvHD and autoantibody expression has been described (18–21). Indeed, autoantibodies may be detectable before the onset of clinical manifestation of cGvHD (15). However, data on the role and clinical implication of autoantibodies in paediatric HSCT recipients are limited.

In this single-centre retrospective study of paediatric patients with ALL, our objectives were to determine autoantibody expression levels following HSCT and investigate whether there was correlation between occurrence of autoantibodies and development of cGvHD, immune reconstitution and survival using serially monitored parameters during long-term follow-up care.

## Materials and Methods

### Patients and Samples

A total of 74 paediatric and adolescent patients with ALL who underwent HSCT at the St. Anna Children's Hospital between February 1993 and June 2020 were included in this retrospective study. Inclusion criteria included: being alive on day +100 after HSCT, complete remission (CR) of the underlying disease, complete multi-lineage donor cell engraftment and no prior treatment with rituximab. Patients' parents' and/or guardians' written informed consent was obtained in accordance with the Declaration of Helsinki and the institutional review board of the Medical University of Vienna and the St. Anna Children's Hospital. All patients underwent HSCT according to standard of care or institutional review board-approved protocols including standard GvHD, antimicrobial and antifungal prophylaxis according to institutional guidelines.

Outpatient post-HSCT care is a calendar-driven at our institution with additional incidence-driven visits, in the event of complications. During routine follow-up visits, where all of these patients were seen in the HSCT Outpatient Clinic of our institution, clinical parameters were collected regarding patient and transplant characteristics and details of GvHD, and peripheral blood samples were analysed for cellular and humoral parameters of immune reconstitution, including analysis of autoantibody panels. Evaluations (clinical and laboratory) were performed at day +100 and every 3–6 months in the first year, every 6 months in the second year, and once a year thereafter and/or as clinically indicated. Acute GvHD (aGvHD) was scored according to the modified Glucksberg criteria (22) and chronic GvHD was graded according to the NIH consensus criteria 2005 (6) and revised 2014 NIH criteria (7). Per definitions, classic cGvHD included classic and overlap subtypes (the presentation of symptoms both of acute and chronic GvHD) and late aGvHD. All outcome data such as overall survival (OS), non-relapse mortality (NRM) and relapse of ALL were retrospectively reviewed for accuracy.

### Laboratory Assessments

Each patient's serum was screened for the presence of autoantibodies, with testing performed either by enzyme-linked immunosorbent assay and/or immunofluorescence. Details of methodologies for antibody assessment are listed in Table 1. For immunoassay-based methods used to screen antibodies, patient values were compared to the relevant reference interval as provided by the manufacturer. Serum titre of ≥1:100 was considered positive and samples were titred at 1:320, 1:640, and 1:1,000. In-house ELISA antibody testing was performed on the DSX ELISA processing system. Patients positive for antinuclear antibody (ANA) were further screened for Smith/anti-ribonucleoprotein (anti-Sm/RNP), anti-Sjögren syndrome-related antigen A (SSA) and anti-Sjögren syndrome type B (SSB) autoantibodies.

**Table 1:** Characteristics of autoantibodies analysed.

**Name**	**Method of detection**	**Kit manufacturer**	**Reference value**	**Units**
Antinuclear antibody	Indirect Immunofluorescence (IIF)	IMMCO	<1:160	
Anti-rheumatoid factor IgG, IgA, or IgM antibody	ELISA	Orgentec	<20	IgG/IgA:U/ml IgM IU/ml
Anti- (single-stranded or) double-stranded DNA antibody	IIF	IMMCO	<1:10	
Anti-alpha-galactosidase antibody	IIF	IMMCO	<1:80	
Anti-mitochondrial M2 antibody	ELISA	Orgentec	<10	U/ml
Anti-mitofilin antibody	IIF	IMMCO	<1:80	
Anti-centromere autoantigen A or alpha-1 antichymotrypsin antibody	ELISA IIF	Orgentec Euroimmun	<10 <1:100	U/ml
Anti-collagen antibody	ELISA	In house		
Anti-beta-2 glycoprotein antibody IgG/IgM	ELISA	Orgentec	<5	U/ml
Anti-Sjögren's syndrome type B antibody	ELISA	Orgentec	<15	U/ml
Anti-Sjögren's-syndrome-related antigen A antibody	ELISA	Orgentec	<15	U/ml
Anti-citron (Rho-interacting serine/threonine kinase) antibody	ELISA	Orgentec	20	U/ml
Anti-Smith antibody	ELISA	Orgentec	<15	U/ml
Anti-ribonucleoprotein antibody	ELISA	Orgentec	<25	U/ml
Anti-exosome antibody (scleroderma)	ELISA	Orgentec	<15	U/ml
Anti-histidyl tRNA synthetase antibody	ELISA	Orgentec	<15	U/ml
Anti-liver-kidney microsomal antibody	ELISA	Orgentec	<11	U/ml
Anti-thyroid antibody	ELISA	Orgentec	TPO <50 TG ≤ 100	IU/ml
Anti-neutrophil cytoplasmic antibody	IIF	INOVA	<1:40	
Anti-liver cytosolic antigen type 1 antibody	ELISA	Orgentec	<11	U/ml
Anti-thrombocyte antibody	Simultaneous analysis of specific platelet antibodies, Luminex (PakLx Assay)	Immucor		pos./neg.
Anti-nucleobindin 1 antibody	ELISA	Orgentec	<20	U/ml
Anti-cardiolipin G/M antibody	ELISA	Orgentec	IgG/IGA <10 IgM <7	U/ml
Anti-complement C3 antibody	Nephelometry	Siemens	90–180	mg/dl

The following assessments on serum were performed during routine follow-up examinations of patients longitudinally: leukocyte subpopulations; blood counts; concentrations of total immunoglobulin (Ig) G, and IgG subclasses 1–4, IgM, IgA, IgE; numbers of specific T-cell subpopulations (CD3^+^, CD4^+^, and CD8^+^ and the ratio of CD4^+^/CD8^+^), natural killer (NK) cells (CD3^−^CD56^+^CD16^+^), and specific B-cell subsets (CD19^+^, CD19^+^CD27^+^ memory, CD19^+^CD27^+^IgD^+^ non-class-switched memory, CD19^+^CD27^+^IgD^−^ class-switched memory, CD19^+^CD21^low^, and the ratio of CD19CD21^low^/CD19^+^CD27^+^). Flow cytometry with gating strategy was described previously and involved the isolation of blood cells, immunophenotyping, flow cytometry, and fluorescence *in situ* hybridisation of sorted cells (9, 17, 23). Optimal concentrations of directly conjugated monoclonal antibodies were added to 50 μL of patients' whole blood and incubated at room temperature for 20 min. ADG lysis solution (An der Grub, Vienna, Austria) was used to remove red blood cells according to the manufacturer's recommendations followed by acquisition of 5 × 10^3^ cells in the lymphogate for leukocyte subpopulations and 4–8 × 10^3^ CD19^+^ B cells for B-cell subset analysis as previously described (9, 17, 23). Serum levels of IgG, IgM, and IgA were quantified by nephelometry using Beckman Coulter IMMAGE (Beckman Coulter Inc., Brea, CA).

### Statistical Analysis

Patients were divided into subgroups: autoantibody positive and autoantibody negative (based on absolute values or titres), and patients with or without cGvHD. Fisher's exact test was used to compare differences in categorical variables. For univariate analyses, different subpopulations and detailed clinical cGvHD characteristics at study points throughout long-term follow-up were selected and compared using the student's *t*-test or the Mann-Whitney *U*-test for continuous variables. Covariates with a *p* < 0.05 were entered into the multiple logistic regression analysis. If absolute values or percent values of a covariate were available as different variables, then these covariates were entered into multivariate logistic regression analysis. OS was calculated from day 0 of HSCT to the day of death from any cause, relapse or last follow-up to 1 January 2021. Patients were censored at the date of last contact. OS was analysed using the Kaplan-Meier test, and both groups were compared using a log-rank test or a Breslow test. NRM was defined as death due to causes unrelated to the underlying disease. Disease relapse and cGvHD were considered competing risks in this analysis. Statistical analyses were performed with SPSS 20.0 software (IBM Company, Chicago, IL, USA). Differences were considered statistically significant at a *p* < 0.05.

## Results

### Patient and Transplant Characteristics and HSCT Outcomes

Between February 1993 and June 2020, 74 patients who received HSCT for ALL were enrolled in the study, yielding 440 serum samples for analysis. Median age was 10.9 years (range 2.7–22.2 years) and median follow-up was 8 years (range 0.4–19.3 years). In this homogenous cohort the distribution of patient and transplant characteristics such as age, sex, conditioning regimen, donor type, stem cell source, and GvHD prophylaxis was similar between patients who did and did not express autoantibodies during follow-up (Table 2).

**Table 2:** Demographic and transplant characteristics and outcomes of paediatric ALL patients after HSCT.

	**All patients**	**Autoantibody positive**	**Autoantibody negative**
	**(*N* = 74)**	**(*n* = 48)**	**(*n* = 26)**
Median age at HSCT, years (range)	10.74 (1.03–23.85)	11.19 (2.67–22.23)	9.08 (1.04–23.85)
Male, *n* (%)	47 (64%)	31 (65%)	16 (62%)
Female, *n* (%)	27 (36%)	17 (35%)	10 (38%)
Conditioning regimen, *n* (%)			
Chemotherapy-based myeloablation	73 (99%)	47 (98%)	26 (100%)
Total-body-irradiation–based myeloablation	67 (91%)	45 (94%)	22 (85%)
Reduced-intensity conditioning	1 (1%)	1 (2%)	0
Stem cell donor, *n* (%)			
Related donor	28 (38%)	18 (37%)	10 (38%)
Matched unrelated donor	18 (24%)	10 (21%)	8 (31%)
Mismatched unrelated donor	28 (38%)	20 (42%)	8 (31%)
Stem cell source, *n* (%)			
Bone marrow	60 (81%)	40 (84%)	20 (77%)
Peripheral blood stem cells	14 (19%)	8 (16%)	6 (23%)
Median number of infused CD34^+^ cells × 10^6^/kg (range)	3.7 (0.9–62)	3 (0.9–62)	4.8 (1.2–17)
GvHD prophylaxis			
Cyclosporine A only	28 (38%)	20 (42%)	8 (31%)
Cyclosporine A + methotrexate	45 (61%)	28 (58%)	17 (65%)
Cyclosporine A + mycophenolate mofetil	1 (1%)	0	1 (4%)
Included anti-thymocyte globulin^*^	39 (53%)	27 (56%)	12 (46%)
Acute GvHD, *n* (%)	56 (76%)	38 (79%)	18 (69%)
Grade II–IV	27 (36%)	18 (38%)	9 (35%)
Grade III–IV	8 (11%)	6 (13%)	2 (8%)
Late acute GvHD, *n* (%)	3 (4%)	2 (4%)	1 (4%)
Chronic GvHD, *n* (%)	18 (24%)	14 (29%)	4 (15%)
Onset type of cGvHD, *n* (%)			
Progressive	7/18 (39%)	5/14 (36%)	2/4 (50%)
Quiescent	10/18 (56%)	8/14 (57%)	2 /4 (50%)
*De novo*	1/18 (5%)	1/14 (7%)	0
NIH classification of cGvHD, *n* (%)			
Classic chronic	10/18 (56%)	9/14 (64%)	1/4 (25%)
Overlap	8/18 (44%)	5/14 (36%)	3/4 (75%)
Overall severity of cGvHD, *n* (%)			
Mild	3/18 (17%)	3/14 (21%)	0
Moderate	2/18 (11%)	2/14 (14%)	0
Severe	13/18 (72%)	9/14 (64%)	4/4 (100%)
Organ involvement of GvHD, *n* (%)			
Skin	14/18 (78%)	12/14 (86%)	2/4 (50%)
Scleroderma	9/18 (50%)	7/14 (50%)	2/4 (50%)
Oral mucosa	10/18 (56%)	8/14 (57%)	2/4 (50%)
Eyes	7/18 (39%)	5/14 (36%)	2/4 (50%)
Joints	5/18 (28%)	4/14 (29%)	1/4 (25%)
Gastrointestinal	3/18 (17%)	2/14 (14%)	1/4 (25%)
Liver	7/18 (39%)	5/14 (36%)	2/4 (50%)
Genital	0	0	0
Lungs	3/18 (17%)	0	3 (75%)
Other	2/18 (11%)	1/14 (7%)	1/4 (25%)
Median duration of cGvHD, months	42.6	36	47.3
10-year OS, *n* (%)	66/74 (89%)	44/48 (92%)	22/26 (85%)
Relapse of ALL, death, *n* (%)	4/74 (5%)	2/48 (4%)	2/26 (8%)
Relapse of ALL, alive, *n* (%)	6/74 (8%)	2/48 (4%)	4/26 (15%)

*P-value generated with Fisher test; all differences were not significant*.

* *Anti-thymocyte globulin was given as part of the conditioning regimen in addition to other compounds. ALL, acute lymphoblastic leukaemia; cGvHD, chronic GvHD; GvHD, graft-versus-host disease; HSCT, haematopoietic stem cell transplantation; NIH, National Institutes for Health; OS, overall survival*.

**Figure 1:**
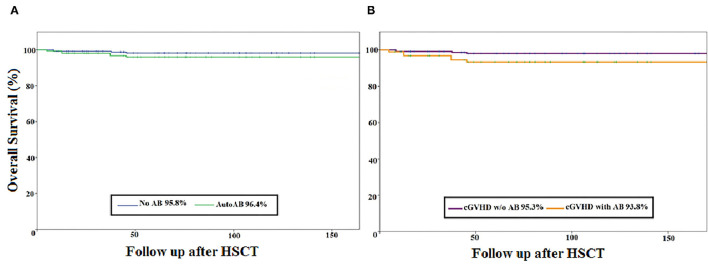
Kaplan–Meier curve of the overall survival of paediatric ALL patients after HSCT (*N* = 74). **(A)** overall survival (percentage) of the autoantibody-negative (*n* = 26) vs. the autoantibody-positive (*n* = 48) patient group. **(B)** Overall survival (percentage) of the patient group with cGvHD (*n* = 21) vs. the patient group without GvHD (*n* = 53). AB, antibody; cGvHD, chronic graft-versus-host disease; HSCT, haematopoietic stem cell transplantation; w/o, without; Follow-up after HSCT: in months.

In the cohort, 81% (60/74) of patients received a bone marrow graft, 62% (46/74) received stem cells from an unrelated donor, and 53% (39/74) received anti-thymocyte globulin as part of their conditioning. aGvHD of grade II–IV and grade III–IV was diagnosed in 36.5% (27/74) and 11% (8/74) of patients, respectively. cGvHD was diagnosed in 24% (18/74) of patients; severe in 72% (13/18), progressive onset in 39% (7/18), and evidence of overlap cGvHD in 44% (8/18). The majority of patients with cGvHD (93%) had a history of aGvHD. The most frequent organs affected by cGvHD were skin (78%), oral mucosa (56%) and eye (39%). Over 90% of patients suffered from multiorgan involvement, identified as cGvHD in ≥2 organs. The relapse rate of ALL was low, at 13.5% (10/74). The 10-year OS was excellent at 89% for the whole cohort. The overall mortality rate was 11% (8/74), with death occurring at a median of 5.5 years after HSCT. No difference in 5-year OS was seen when comparing the antibody-positive and the antibody-negative group (96.4 vs. 95.8%, respectively) and no influence of cGvHD was observed (95.3 vs. 93.8%, respectively) (Figures 1A,B). Causes of death were relapse of ALL in 4 patients, cGvHD in 1 patient, secondary malignancy in 1 patient, infection in 1 patient, and sudden death with epilepsy and brain oedema as determined by autopsy in 1 patient. Of note, a history of cGvHD was evident in 4 of 8 patients, but none of the patients who died of ALL relapse had a history of cGvHD.

### Clinical Outcomes in Patients With and Without Autoantibodies

At least one type of autoantibody was detected in 65% (48/74) patients during the follow-up period. Antinuclear antibodies were the most frequently detected antibody type, occurring in 75% (36/48) of those patients with autoantibodies (Table 2). The incidence of aGvHD was similar between groups, even when analysed by grade. Likewise, the incidence of cGvHD was comparable between groups, indicating that cGvHD was not associated with autoantibody production in our cohort. Although not statistically significant due to the low sample sizes, NIH-defined classic cGvHD was overrepresented in the autoantibody-positive group vs. the autoantibody-negative group (64 vs. 25%, respectively). Conversely, overlap manifestations of cGvHD at onset were more often diagnosed in the autoantibody-negative group than the autoantibody-positive group (75 vs. 36%, respectively). No significant correlation was found between autoantibody positivity and NIH-defined overall severity of cGvHD; however, all 4 patients (100%) in the autoantibody-negative group with cGvHD had severe cGvHD in comparison to 9 out of the 14 (64%) patients in the autoantibody-positive group with cGvHD. With regard to the organ involvement of cGvHD, no association with autoantibody expression was observed.

There were no significant differences in mortality between the autoantibody-positive group (8%, 4/47) and the autoantibody-negative group (15%, 4/47; *p* = 0.44). Neither the expression of autoantibodies nor the occurrence of cGvHD was correlated with significantly worse survival (Figures 1A,B). The relapse rate of ALL did not differ significantly between the two groups, being 8% (4/48) for the autoantibody-positive group and 23% (6/26) for the autoantibody-negative group (Table 2).

### Prevalence and Type of Autoantibodies and cGvHD

At least one type of autoantibody was detected in 65% (48/74) of patients and multiple autoantibodies were detected in 36% (27/74) of patients (Table 3). Antinuclear antibodies were the most frequently detected antibody type, occurring in 75% (36/48) of autoantibody-positive patients. Of patients with autoantibodies, 33% (16/48) had a history of cGvHD (including 14 cases of classic chronic and 2 cases of late aGvHD, as shown in Table 2). Of patients with autoantibodies and cGvHD, the most common antibody type was antinuclear antibody (88%, 14/16), followed by anti-rheumatoid factor antibody (38%) and anti-collagen antibody (25%). There was a trend towards patients with cGvHD being more likely to express antinuclear antibody than patients without cGvHD, although this was not statistically significant [67 (14/21) vs. 41% (22/53), respectively; *p* = 0.07]. Due to the small number of patients, no statistical analysis of the correlation between specific autoantibodies and the organ involvement of cGvHD during long-term follow-up was possible (descriptive data are shown in Table 4).

**Table 3:** Prevalence of autoantibodies during long-term follow-up in the patient group without (no) cGvHD and with cGvHD.

**Autoantibody positive, *n* (%)**	**No GvHD**	**cGvHD**	***P*-value**
	**(*n* = 32)**	**(*n* = 16)**	
Any autoantibody	32 (60%)	16 (76%)	0.28
Antinuclear antibody	22 (69%)	14 (88%)	0.07
Anti-rheumatoid factor IgG, IgA, or IgM antibody	13 (41%)	6 (38%)	ns
Anti-single-stranded or double-stranded DNA antibody	2 (6%)	0	ns
Anti-alpha-galactosidase antibody	9 (28%)	1 (6%)	ns
Anti-mitochondrial M2 antibody	1 (3%)	1 (6%)	ns
Anti-mitofilin antibody	1 (3%)	0	ns
Anti-centromere autoantigen A or alpha-1 antichymotrypsin antibody	3 (9%)	2 (13%)	ns
Anti-collagen antibody	3 (9%)	4 (25%)	ns
Anti-beta-2 glycoprotein antibody	1 (3%)	3 (19%)	ns
Anti-Sjögren's syndrome type B antibody	1 (3%)	1 (6%)	ns
Anti-Sjögren's-syndrome-related antigen A antibody	1 (3%)	0	ns
Anti-citron (Rho-interacting serine/threonine kinase 21) antibody	1 (3%)	1 (6%)	ns
Anti-Smith antibody	1 (3%)	0	ns
Anti-ribonucleoprotein antibody	2 (6%)	0	ns
Anti-exosome antibody (scleroderma)	1 (3%)	0	ns
Anti-histidyl tRNA synthetase antibody	1 (3%)	0	ns
Anti-liver-kidney microsomal antibody	1 (3%)	0	ns
Anti-thyroid antibody	1 (3%)	2 (13%)	ns
Anti-neutrophil cytoplasmic antibody	2 (6%)	1 (6%)	ns
Anti-liver cytosolic antigen type 1 antibody	1 (3%)	0	ns
Anti-thrombocyte antibody	1 (3%)	0	ns
Anti-nucleobindin 1 antibody	1 (3%)	0	ns
Anti-cardiolipin G/M antibody	1 (3%)	0	ns
Anti-complement C3 antibody	0	1 (6%)	ns

*P-value generated with Fisher test. cGvHD, chronic graft-versus-host disease; Ig, immunoglobulin; ns, not significant*.

**Table 4:** Prevalence of autoantibodies in relation to organ involvement of cGvHD in 16 patients^*^.

	**cGvHD organ involvement**				
**Autoantibody positive, *n* (%)**	**Skin**	**Scleroderma and joints**	**Liver**	**Eyes**	**Oral**
Antinuclear antibody	12 (75%)	8 (50%)	4 (25%)	5 (31%)	8 (50%)
Anti-rheumatoid factor IgG, IgA, or IgM antibody	4 (25%)	1 (6%)	3 (19%)	1 (6%)	4 (25%)
Anti-alpha galactosidase antibody	1 (6%)	1 (6%)	1 (6%)	1 (6%)	1 (6%)
Anti-mitochondrial M2 antibody	1 (6%)	1 (6%)	1 (6%)	1 (6%)	1 (6%)
Anti-centromere autoantigen A or alpha-1 antichymotrypsin AB	2 (13%)	1 (6%)	1 (6%)	0	0
Anti-collagen antibody	3 (19%)	1 (6%)	3 (19%)	1 (6%)	3 (19%)
Anti-beta-2 glycoprotein antibody	2 (13%)	1 (6%)	2 (13%)	1 (6%)	1 (6%)
Anti-Sjögren's syndrome type B antibody	1 (6%)	1 (6%)	1 (6%)	1 (6%)	1 (6%)
Anti-citron (Rho-interacting serine/threonine kinase 21) antibody	1 (6%)	1 (6%)	0	0	1 (6%)
Anti-thyroid antibody	2 (13%)	0	1 (6%)	0	0
Anti-neutrophil cytoplasmic antibody	1 (6%)	1 (6%)	1 (6%)	1 (6%)	0
Anti-complement C3 antibody	1 (6%)	0	0	1 (6%)	0

* *14 patients had classic chronic GvHD and 2 had late acute GvHD. cGvHD, chronic graft-versus-host disease; Ig, immunoglobulin*.

### Comparison of Longitudinally Assessed Humoral and Cellular Parameters of Patients With Autoantibodies vs. Those Without Autoantibodies

To assess immunological disparities between autoantibody-positive and negative groups, we compared the humoral and cellular parameters of 440 blood samples collected during long-term follow-up at consecutive time points alongside concurrent clinical data. In autoantibody-positive patients we found significantly increased mean numbers of leukocytes (6,410 vs. 5,815 × 10^3^ cells/mL, respectively; *p* = 0.039), granulocytes (3,736 vs. 3,341 × 10^3^ cells/mL, respectively; *p* = 0.034), lymphocytes (2,160 vs. 1,910 × 10^3^ cells/mL, respectively; *p* = 0.024), and monocytes (504.2 vs. 437.7 × 10^3^ cells/mL, respectively; *p* = 0.005), as shown in Table 5. Furthermore, the prevalence of autoantibodies was associated with significantly higher numbers of T cells and B cells including CD3^+^ T cells (1,508 vs. 1,276 × 10^3^, respectively; *p* = 0.023), CD8^+^ T cells (720.8 vs. 616.6 × 10^3^, respectively; *p* = 0.05), and CD19^+^ B cells (507.2 vs. 335.6 × 10^3^, respectively; *p* = 0.006). Similarly, significantly higher mean immunoglobulin concentrations–such as IgG (1,080 vs. 896.1 mg/dL, respectively; *p* < 0.001), IgG3 (77.3 vs. 63.9 mg/dL, respectively; *p* = 0.021), IgG4 (43.8 vs. 36.7 mg/dL, respectively; *p* = 0.05), and IgM (114.9 vs. 86.5 mg/dL, respectively; *p* = 0.009) were observed in autoantibody-positive vs. autoantibody-negative patients.

**Table 5:** Longitudinal assessment of humoral and cellular parameters of patients with autoantibodies vs. those without autoantibodies.

**Parameter**	**Autoantibody positive**	**Autoantibody negative**	***P*-value**
Mean numbers of immune cells × 10^3^ cells/mL			
Leukocytes	6,448.8	5,700.6	0.002
Lymphocytes	2,159.5	1,910.1	0.001
Monocytes	504.2	437.7	0.005
Granulocytes	3,715.4	3,299	0.002
CD3^+^ T cells	1,507.7	1,276.3	0.016
CD8^+^CD3^+^T cells	720.8	616.6	0.013
CD19^+^ B cells	507.2	335.6	0.006
Mean concentrations of immunoglobulins, mg/dL			
IgG	1,079.9	896.1	0.000
IgM	114.9	86.5	0.009
IgG3	77.3	63.9	0.047
IgG4	43.8	36.7	0.050

*Differences between groups were compared using the student's t-test or the Mann-Whitney U-test for continuous variables. Ig, immunoglobulin*.

### Autoantibody Expression, Immune Reconstitution, and Presence of cGvHD

In samples positive for autoantibodies, mean numbers of leukocytes (6.534 vs. 4.852 × 10^3^ cells/mL, *p* < 0.001), granulocytes (3.739 vs. 3.010 × 10^3^ cells/mL, respectively; *p* = 0.018), lymphocytes (2.225 vs. 1.422 × 10^3^ cells/mL, respectively; *p* = 0.024), and monocytes (504.1 vs. 363.0 × 10^3^ cells/mL, respectively; *p* = 0.001) were higher for patients with cGvHD vs. those without cGvHD (Table 6). The significantly higher numbers of immune cells in cGvHD patients with autoantibodies involved CD4^+^ T cells (mean 632.6 vs. 384.0 × 10^3^ cells/mL, *p* < 0.0001), CD8^+^ T cells (759.3 vs. 447.2 × 10^3^ cells/mL, respectively; *p* < 0.001), CD19^+^ B cells (539.6 vs. 276.3 × 10^3^ cells/mL, *p* = 0.003) and CD56^+^ NK cells (236.12 vs. 165.6 × 10^3^ cells/mL, *p* = 0.023). Moreover, autoantibody production in cGvHD patients was associated with significantly higher mean numbers of CD21^low^ B cells (23.8 vs. 11.8 × 10^3^ cells/mL, *p* = 0.044), and a distorted ratio of CD21^low^ B cells/CD27^+^ memory B cells (1.7 vs. 0.4 respectively; *p* = 0.006). Elevated levels of immunoglobulins such as IgG (1,159 vs. 916.9 mg/dL, respectively, *p* = 0.009), IgG3 (77.4 vs. 45.8 mg/dL, respectively; *p* < 0.001), and IgM (129.8 vs. 88.0 mg/dL, respectively; *p* = 0.038). Multivariate logistical regression analysis showed that increase of CD8^+^ T cells (*p* = 0.03), CD56^+^ NK cells (*p* = 0.04), and IgG3 (*p* = 0.043) was significantly associated with antibody production in cGvHD patients.

**Table 6:** Longitudinal assessment of humoral and cellular parameters of patients with autoantibodies both with and without cGvHD.

**Parameter**	**Autoantibody positive with cGvHD**	**Autoantibody positive without cGvHD**	***P*-value**
Mean numbers of immune cells × 10^3^ cells/mL			
Leukocytes	6,534.1	4,852.4	0.000
Lymphocytes	2,224.7	1,422.3	0.000
Monocytes	504.1	363.0	0.001
Granulocytes	3,738.7	3,009.6	0.018
CD56^+^CD3^+^ NK cells	236.1	165.6	0.023
CD3^+^ T-cells	1,501.5	1,364.2	0.005
CD4^+^CD3^+^T cells	632.6	384.0	0.000
CD8^+^CD3^+^T cells	759.3	447.2	0.000
CD19^+^ B cells	539.6	276.3	0.003
CD19^+^CD21^low^ B cells	23.8	11.8	0.044
Ratio CD21^low^ B cells/CD27^+^B cells	1.7	0.4	0.006
Mean concentrations of immunoglobulins, mg/dL			
IgG	1,159.3	916.9	0.009
IgM	129.8	88.0	0.038
IgG3	77.4	45.8	0.000

*Differences between groups were compared using the student's t-test or the Mann-Whitney U-test for continuous variables. cGvHD, chronic graft-versus-host disease; Ig, immunoglobulin*.

It has been previously demonstrated that autoantibody production may correlate with activity of cGvHD. Therefore, we assessed the correlation between autoantibody expression in patients with precisely assessed *active* cGvHD at the time samples were taken. Results mirrored the above-reported significant association between immunological parameters and cGvHD (data not shown). In addition, a significantly diminished proportion of CD27^+^ memory B cells (9.1 vs. 14.9%, respectively; *p* = 0.015) and an aberrantly low ratio of class-switched CD27^+^IgD^−^/CD27^+^ memory B cells (3.5 vs. 5.1, respectively; *p* = 0.013) were observed in patients with *active* cGvHD expressing autoantibodies, suggestive of B-cell perturbation (Table 7).

**Table 7:** Assessment of humoral and cellular parameters at the time of analysis in patients with autoantibodies both with and without *active* cGvHD.

**Parameter**	**Autoantibody positive with *active* cGvHD**	**Autoantibody positive without *active* cGvHD**	***P*-value**
CD19^+^21^low^ B cells, × 10^3^ cells/mL	36.8	11.8	0.013
Ratio CD19^+^CD21^low^ B cells/CD27^+^B cells	2.4	0.4	0.034
CD19^+^CD27^+^ B cells, %	9.1	14.9	0.028
CD19^+^CD27^+^IgD^−^, %	3.5	5.1	0.013

*Differences between groups were compared using the student's t-test or the Mann-Whitney U-test for continuous variables. cGvHD, chronic graft-versus-host disease*.

To assess the potential association between autoantibody production and the number of CD21^low^ B cells, which are a well-known marker of B-cell perturbation in cGvHD, we analysed the distribution of CD21^low^ B cells using a cutoff >7% based on the publication by Wehr et al. (24). In 3 assessments the proportion of CD21^low^ B cell was >7%. About 19 of 93 assessments were derived from the autoantibody-positive group and 74/93 assessments were derived from the autoantibody-negative group. When considering the *activity* of cGvHD at the time the samples were taken, 9 of the 19 CD21^low^ B-cell samples that were derived from autoantibody-positive patients were from patients with *active* cGvHD (47%), while 61 of 74 CD21^low^ B-cell samples that were derived from autoantibody-negative patients were from patients with *active* cGvHD (82%) (*p* = 0.0053).

To determine whether cGvHD is the main reason for the significant impairment of immune homeostasis regardless of the autoantibody expression, we compared cellular and humoral parameters in patients with cGvHD at any study time point to a homogenous cohort of patients without cGvHD, as shown in Table 8. This analysis was independent of autoantibody expression. cGvHD was significantly associated with increased mean numbers of leukocytes (6.289 vs. 4.878 × 10^3^ cells/mL, *p* < 0.0001), granulocytes (3.603 vs. 2.922 × 10^3^ cells/mL, respectively; *p* < 0.001), lymphocytes (2.154 vs. 1.476 × 10^3^ cells/mL, respectively; *p* < 0.0001), and monocytes (489.9 vs. 365.4 × 10^3^ cells/mL, respectively; *p* < 0.0001). cGvHD was also associated with significantly higher numbers of CD3^+^ T cells (1.445 vs. 1.086 × 10^3^ cells/mL, respectively; *p* = 0.008), CD4^+^ T cells (619.7 vs. 476.1 × 10^3^ cells/mL, respectively; *p* < 0.0001), CD8^+^ T cells (717.9 vs. 446.6 × 10^3^ cells/mL, respectively; *p* < 0.001), and CD56^+^ NK cells (262.3 vs. 166.1 × 10^3^ cells/mL, respectively; *p* < 0.001) as well as a diminished CD4/CD8 ratio (1.1 vs. 1.2, *p* = 0.017). In cGvHD vs. no cGvHD patients, the B-cell compartment showed significantly increased CD19^+^ B cells (430.9 vs. 295.5 × 10^3^ cells/mL, respectively; *p* < 0.0001) and CD21^low^ B cells (9.7 vs. 6.6%; *p* = 0.019), together with elevated immunoglobulin levels (IgG: 993.0 vs. 895.3 mg/dL, *p* = 0.022; IgG1: 687.7 vs. 580.7 mg/dL; *p* = 0.014; IgG3: 75.0 vs. 51.8 mg/dL; *p* < 0.0001, respectively).

**Table 8:** Longitudinal assessment of humoral and cellular parameters of patients with and without (no) cGvHD independently of autoantibody expression.

**Parameter**	**cGvHD**	**No cGvHD**	***P*-value**
Mean numbers of immune cells × 10^3^ cells/mL			
Leukocytes	6,289.4	4,878.0	0.000
Lymphocytes	2,154.2	1,476.1	0.000
Monocytes	489.9	365.4	0.000
Granulocytes	3,602.9	2,921.8	0.000
CD56^+^3^−^ NK cells	262.3	166.1	0.000
CD3^+^ T cells	1,444.9	1,086.3	0.008
CD4^+^3^+^ T cells	619.7	476.1	0.000
CD8^+^3^+^ T cells	717.9	446.6	0.000
CD19^+^ B cells	430.9	295.5	0.000
CD19^+^21^low^ B cells, %	9.7	6.6	0.019
CD19^+^21^low^ B cells	31.0	17.9	0.000
Ratio CD4^+^/CD8^+^ T cells	1.1	1.2	0.017
Ratio CD21^low^B cells/CD27^+^ B cells	1.6	1.3	0.013
Mean concentrations of immunoglobulins, mg/dL			
IgG	993.0	895.3	0.022
IgG1	687.7	580.7	0.014
IgG3	75.0	51.8	0.000

*Differences between groups were compared using the student's t-test or the Mann-Whitney U-test for continuous variables. cGvHD, chronic graft-versus-host disease; Ig, immunoglobulin*.

### Association Between Survival and Parameters of Immune Reconstitution

When comparing survivors vs. non-survivors (Table 9) survivors showed significantly higher mean numbers of CD4^+^ T cells (595.1 vs. 440.0 × 10^3^ cells/mL, respectively; *p* = 0.04), with a normalised CD4/CD8 ratio (1.1 vs. 0.8 × 10^3^ cells/mL, respectively; *p* = 0.041). Additionally, in survivors the B-cell compartment revealed a tendency towards normalisation regarding CD19^+^ B cells (406.1 vs. 289.4 × 10^3^ cells/mL respectively; *p* = 0.042), CD27^+^ B memory cells (54.9 vs. 30.6 × 10^3^ cells/mL, respectively; *p* = 0.05), mainly class-switched CD27^+^IgD^−^ B cells (21.2 vs. 5.0 × 10^3^ cells/mL, respectively; *p* < 0.0001), the ratio of CD27^+^IgD^+^/CD27^+^IgD^−^ B cells (2.2 vs. 5.9, *p* = 0.003), and IgG4 (40.9 vs. 19.4 mg/dL, respectively; *p* < 0.0001). In contrast, non-survival was associated with a significantly elevation of the proportion of CD21^low^ B cells (13.4 vs. 8.8%, *p* = 0.039), and CD56^+^ NK cells (238.8 vs. 314.1, × 10^3^ cells/mL, respectively; *p* = 0.019). In multivariate analysis, greater survival was significantly associated with lower mean numbers of CD56^+^ NK cells [hazard ratio (HR) 0.98, *p* = 0.041] and higher mean numbers of CD27^+^ memory B cells (HR 1.62, *p* = 0.014).

**Table 9:** Longitudinal assessment of humoral and cellular parameters in survivors vs. non-survivors independently of autoantibody expression.

**Parameter**	**Survivors**	**Non-survivors**	***P*-value**
Mean numbers of immune cells × 10^3^ cells/mL			
CD56^+^3^−^ NK cells	238.8	314.1	0.019
CD4^+^3^+^ T cells	595.1	440.0	0.043
CD19^+^ B cells	406.1	289.4	0.042
CD19^+^CD27^+^ B cells	54.9	30.6	0.050
CD27^+^ IgD^−^ B cells	21.2	5.0	0.000
CD19^+^21^low^ B cells, %	15.3	30.1	0.002
Ratio CD4^+^/CD8^+^T cells	1.1	0.8	0.041
Ratio CD27^+^ IgD^+^/CD27+IgD^−^ B cells	2.2	5.9	0.003
Mean concentration of IgG4, mg/dL	40.9	19.4	0.000

*Differences between groups were compared using the student's t-test or the Mann-Whitney U-test for continuous variables. Ig, immunoglobulin*.

## Discussion

Dysregulated immunity in cGvHD might be comparable to autoimmune diseases, where the pathogenic role of autoantibodies has been similarly shown in adult (12, 14, 15) and paediatric HSCT patients (25). This study aimed to determine the prevalence and potential value of autoantibodies as cGvHD biomarkers in the context of immune reconstitution in paediatric ALL patients after HSCT.

In this homogenous study cohort, we detected autoantibodies in 65% of patients, higher than in recently published adult cohorts (18, 21). When comparing demographic data and transplant characteristics of patients with and without autoantibody expression there were no significant differences. Although the prevalence of autoantibodies was greater in patients with cGvHD than in those without cGvHD (76 vs. 60%, respectively), this difference was not significant. This may be due to sample size. Furthermore, we did not find a significant association between the prevalence of autoantibodies and the overall severity of cGvHD, which is consistent with previous findings in adults (15, 18). Of note, in this cohort all 4 autoantibody-negative patients who had cGvHD suffered from severe cGvHD.

Consistent with previous studies (15, 18, 20), antinuclear antibodies were the most common autoantibody type detected in our patients. While cGvHD patients had a higher frequency of antinuclear autoantibodies than did patients without cGvHD (88 vs. 79%, respectively; *p* = 0.07), this difference did not reach statistical significance. This is in contrast to findings by Patriarca et al. (20) and Yang et al. (21) in adult HSCT patients and might possibly be explained by the lower incidence of cGvHD in paediatric patients. Further analyses regarding organ involvement of cGvHD and autoantibody profiles in our cohort were hampered by the low case numbers. In contrast to adult studies (18, 21), autoantibody profiles linked to systemic lupus erythematosus (SLE) and systemic sclerosis (such as anti-single-stranded or double-stranded DNA, anti-SSB and anti-SSA antibodies) did not correlated with cGVHD in our study. Similarly to published data (15), autoantibody positivity and profiles did not correlate with the severity, activity, or clinical characteristics of cGvHD (data not shown), indicating that autoantibodies are not suitable biomarkers for monitoring cGvHD. Besides, a correlation between explicit autoantibody profiles and specific tissue damage in cGVHD remains to be established.

In a study of 121 adolescent and adult HSCTs (mean age 35 years, range 15–68) for malignant diseases, Moon et al. showed favourable outcome regarding relapse rate and survival in patients with autoantibody positivity (19). Our data showed acceptable relapse and excellent OS with no association between presence of autoantibodies and relapse. Furthermore, neither the expression of autoantibodies nor the occurrence of cGvHD was associated with significantly worse survival (Figures 1A,B). Patients with autoantibodies showed significantly better immune reconstitution, with overall higher numbers of T cells and B cells and higher serum immunoglobulin concentrations, similarly to those reported by Patriarca et al. (20).

Notably, the prevalence of autoantibodies in patients with cGvHD correlated with better immune reconstitution and elevated numbers of NK cells. The association between elevated numbers of NK cells and cGvHD has been described by our group previously in an observational paediatric study in 146 HSCT patients (mean age 8.6 years, range 0.4–19.3 years) with 659 samples during longitudinal follow-up (17). Huenecke et al. reported a similar association between NK-cell reconstitution and cGvHD in a paediatric single centre study of 74 HSCT patients with malignant diseases (26). Among the cGvHD patients in our present study, autoantibody positivity (vs. negativity) was associated with signs of B-cell perturbation, such as significantly higher mean numbers of CD21^low^ B cells (23.8 vs. 11.8 × 10^3^ cells/mL, *p* = 0.044) and a distorted ratio of CD21^low^ /CD27^+^ memory B cells (1.7 vs. 0.4, *p* = 0.006). When considering the *activity* of cGvHD in the autoantibody-positive group, we could not only confirm aberrant B-cell homeostasis but also further strengthen these findings by observing a significantly lower proportion of CD27^+^ memory B cells (9.1 vs. 14.9%, *p* = 0.015) with an altered ratio of class-switched CD27^+^IgD^−^/CD27^+^ memory B cells (3.5 vs. 5.13, *p* = 0.013) in comparison to the autoantibody-positive group without cGvHD. This is consistent with previous studies and reviews that have described disfunctional B-cell homeostasis in patients with cGvHD and the proposed a role of CD21^low^ B cells (9, 17, 27–29). To explore this further, we assessed the potential role of autoantibody expression and CD21^low^ B cells in cGvHD *activity* and, to our surprise, we found a significant correlation between *active* cGvHD and expanded numbers of CD21^low^ B cells (cutoff >7%) in autoantibody-negative vs. autoantibody-positive patients (82 vs. 47%, respectively; *p* = 0.0053). As reported by Hao et al. from a study in 65 adult HSCT patients, these findings may further suggest a minor role of autoantibody profiles in paediatric patients with active cGvHD and signs of B-cell perturbation.

Because our results might suggest that cGvHD is the main reason for a significant impairment of immune homeostasis, we evaluated immune parameters comparing the group with and without cGvHD independently of autoantibody status. We confirmed published adult data (9, 15) that cGvHD is associated with significantly elevated T cell subsets, especially CD4^+^ T cells, and NK cells. We also showed that paediatric cGVHD, similar to adult cGVHD (9, 15) is associated with significantly increased CD21^low^ B cells and elevated immunoglobulin levels as signs of B-cell perturbation. Previously, our group reported that cGVHD in children correlated with low numbers of CD27^+^ memory B cells in a prospective study, and we hypothesised that this subpopulation may serve as a risk factor/marker of cGvHD (17). In this here presented retrospective study we could not confirm this, emphasising on the need for further prospective multicentre studies on B-cell subpopulations in paediatric HSCT for malignant diseases in correlation with cGvHD.

The expression of circulating autoantibodies as a prognostic marker of survival or relapse has been investigated, which we could not confirm as described above. Therefore, we investigated the immune parameters of survivors vs. non-survivors. Among survivors, data revealed normalisation within the B-cell compartment with significantly higher numbers of both CD27^+^ memory B cells and class-switched CD27^+^IgD^−^ B cells together with a mean higher concentration of IgG4 in comparison to non-survivors. Overall mortality was significantly associated with an elevated proportion of CD21^low^ B cells and of CD56^+^ NK cells. In the multivariate analysis, better survival rates were significantly associated with lower numbers of CD56^+^ NK cells (HR 0.98, *p* = 0.041) and higher mean numbers of CD27^+^ memory B cells (HR 1.62, *p* = 0.014). To our knowledge these associations have not been described previously in paediatric ALL patients after HSCT.

Other strengths of the study are the homogenous cohort (paediatric ALL patients with similar HSCT characteristics) with thorough characterisation of NIH-defined cGvHD manifestations during lon-term follow-up. The assessment of cGvHD *activity* at the time of sample taking will allow a better understanding of the disease course of paediatric cGVHD, and may help the clinician in the future to calibrate the intensity of the immunosuppressive treatment.

Our study has several potential limitations. First, it was a retrospective study with low numbers of paediatric cGvHD patients. Secondly, details and intensity of immunosuppressive treatments that patients received were not evaluated. Data on the presence of autoantibodies both of patients and donors prior HSCT would have been informative.

In conclusion, we confirm that autoantibody profiles are not a suitable biomarker for diagnosis of paediatric cGVHD and for prediction of severe forms of this disease. However, this study identified a number of indicators of aberrant immune homeostasis associated with *active* cGvHD in paediatric patients with ALL who underwent HSCT, confirming results of adult studies and in line with our previous results. These indicators may serve as a surrogate measure for a better characterisation of clinical phenotypes of cGvHD. Our findings provide candidate B-cell subpopulations that could be potential targets of cGvHD treatment in paediatric HSCT for underlying malignant diseases and should be evaluated in larger, multicentre studies.

## Data Availability Statement

The raw data supporting the conclusions of this article will be made available by the authors, without undue reservation.

## Ethics Statement

The studies involving human participants were reviewed and approved by Medical University of Vienna St. Anna Children's Hospital. Written informed consent to participate in this study was provided by the participants' legal guardian/next of kin.

## Author Contributions

AL designed the study. LR, MR, NZ, and ZK analysed the data. AL, DB, LR, and ZK wrote the manuscript. WP and RG performed flow cytometric analyses. WP critically revised the manuscript. All authors approved the final version of the manuscript to be submitted.

## Funding

This study was supported in part by the Jubiläumsfonds der Österreichischen Nationalbank (the Anniversary Fund of the Austrian National Bank), number 13215.

## Conflict of Interest

AL has served on advisory boards and received speaker's fees for participation in scientific meetings of the companies Therakos and Novartis. The remaining authors declare that the research was conducted in the absence of any commercial or financial relationships that could be construed as a potential conflict of interest. The handling editor declared a past co-authorship with one of the authors AL.

## Publisher's Note

All claims expressed in this article are solely those of the authors and do not necessarily represent those of their affiliated organizations, or those of the publisher, the editors and the reviewers. Any product that may be evaluated in this article, or claim that may be made by its manufacturer, is not guaranteed or endorsed by the publisher.
